# Effect of TiO_2_ rutile nanorods on the photoelectrodes of dye-sensitized solar cells

**DOI:** 10.1186/1556-276X-8-37

**Published:** 2013-01-19

**Authors:** Young Hee Jung, Kyung-Hee Park, Jeong Seok Oh, Do-Heyoung Kim, Chang Kook Hong

**Affiliations:** 1 School of Applied Chemical Engineering, Chonnam National University, Gwangju, 500-757, South Korea; 2 The Research Institute for Catalysis, Chonnam National University, Gwangju, 500-757, South Korea; 3 Materials Development Center, R&D Division for Hyundai Kia Motors, Hwaseong, Gweonggi-do, 445-130, South Korea

**Keywords:** One-dimensional TiO_2_ nanorods, Photoelectrode, Electron transfer, Dye-sensitized solar cells

## Abstract

In order to enhance the electron transport on the photoelectrodes of dye-sensitized solar cells, one-dimensional rutile nanorods were prepared using electrospun TiO_2_ nanofibers. The grain size of the nanorods increased with increasing temperature. Electrochemical impedance spectroscopy measurements revealed reduced interface resistance of the cells with the one-dimensional rutile nanorods due to the improved electron transport and the enhanced electrolyte penetration. Intensity-modulated photocurrent/photovoltage spectroscopy showed that the one-dimensional rutile nanorods provided the electrons with a moving pathway and suppressed the recombination of photogenerated electrons. However, an excessive quantity of rutile nanorods created an obstacle to the electrons moving in the TiO_2_ thin film. The photoelectrode with 7 wt.% rutile nanorods optimized the performance of the dye-sensitized solar cells.

## Background

One-dimensional (1-D) structured TiO_2_ nanorods show improved electrical and optical properties in the photoelectrodes of dye-sensitized solar cells (DSSCs) [1]. They can provide straight moving paths for electrons and reduce the *e*^−^/h^+^ recombination [2-4]. Further, they scatter sunlight so that the incident light stays longer in the cell [5]. As these properties enhance the solar energy conversion efficiency, much research into the effects of the 1-D structured TiO_2_ on the photoelectrode have been conducted [6-8].

In principle, photoexcited electrons from dye molecules move on a TiO_2_ nanocrystal undergoing a series of trapping and de-trapping events during diffusion. The 1-D nanorods, which are densely packed TiO_2_ nanoparticles, could act as a single crystal and be involved in rapid electron transport, thereby reducing the chances for electron recombination. Furthermore, the TiO_2_ film with random packing of 1-D rods helps the electrolyte to penetrate into the photoelectrode because of the porosity [9,10]. The enhanced interpenetration of electrolyte leads to the dye regeneration by redox process of the electrolyte and enhances the energy conversion efficiency with improved photocurrent.

Few grain boundaries in the TiO_2_ nanorods induce fast electron transport and decrease the electron recombination due to the reduced number of trapping sites in the interfaces [11]. In order to reduce grain boundaries in the nanorods, the crystal size should be increased. TiO_2_ crystal structure (anatase and rutile) and size can be controlled by sintering temperature. The anatase phase has been reported to be developed at temperatures below 800°C, and above the temperatures, it transforms to the more stable rutile phase [12]. Also, the TiO_2_ nanorods sintered at a high temperature have high crystallinity, meaning reduced grain boundaries and decreased trap sites. Electrons moving through the rutile structure undergo less stress because of the reduced number of trap sites on the grain boundaries [13,14]. In addition, the transported electrons can easily migrate from the rutile to anatase phase [15,16]. As the conduction band of the pure anatase phase is typically 0.2 eV more negative than that of the rutile phase, photoexcited electrons injected into the rutile phase migrate to the conduction band of the anatase phase, before passing through the external circuit. The resulting synergistic effects between the anatase and rutile phases lead to energetic electron flows and enhanced photocurrents [17-19].

However, even though the rutile 1-D nanorods provide the electrons with a better moving path and improve electrolyte penetration, a large number of rutile phases simultaneously can become a barrier for electron transport [8]. The increased amount of rutile phase increases the probability of the moving electrons facing a higher energy level, which increases the internal resistance.

In this study, in order to make photoelectrodes with the 1-D rutile nanorods, the electrospun TiO_2_ nanofibers were sintered at various temperatures. The photoelectrodes considerably improved the DSSC energy conversion efficiency, depending on the amount of TiO_2_ nanorods. The intensity-modulated photocurrent spectroscopy, intensity-modulated photovoltage spectroscopy, charge-transfer resistance, and *I*-*V* characteristics of the DSSCs were investigated in order to study the effects of the rutile TiO_2_ nanorods on the cell performance. The purpose of this study is to investigate the effects of the crystal size and amount of the rutile TiO_2_ nanorods on the electron transport in the photoelectrodes of dye-sensitized solar cells.

## Methods

### Preparation of electrospun nanorods

Three grams of polyvinylpyrrolidone (PVP K90, *M*_W_ = 130,000) was dissolved in 27 g of ethanol (Daejung Chemical & Metal Co., Ltd., Shiheung, South Korea), while the TiO_2_ precursor was prepared by adding 12 ml of acetic acid (Kanto Chemical Co., In., Tokyo, Japan) and 12 ml of ethanol into 6 ml of titanium(IV) isopropoxide (Junsei Chemical Co., Ltd., Tokyo, Japan), successively. The solutions were mixed and stirred for 12 h to obtain homogeneity. The solution was loaded into a syringe (SGE Analytical Science, Ringwood, Victoria, Australia) under an applied voltage of 9 kV. TiO_2_ nanofibers were electrospun on Al foil. The spinning rate was controlled by a syringe pump (KDS-100, KD Scientific, Holliston, MA, USA) at 2 ml/h. The tip-to-collector distance was maintained at 20 cm. The obtained TiO_2_ nanofibers were calcined at 450°C, 650°C, 750°C, 850°C, and 1,000°C.

Transmission electron microscopy (TEM) was used to examine the TiO_2_ nanorods, and the crystal structures were characterized by X-ray diffraction (XRD).

### Fabrication of DSSCs with the TiO_2_ nanorods

The ground nanorods, sintered at 450°C, 650°C, 750°C, 850°C, and 1,000°C, were mixed into a homemade TiO_2_ (P25, Degussa-Hüls, Frankfurt/Main, Germany) paste at a loading of 3 wt.% as a preliminary experiment in order to choose the best nanorod. The ground nanorods sintered at 850°C were chosen and mixed into a commercial TiO_2_ anatase paste (Dyesol, Queanbeyan, New South Wales in Australia) at ratios of 0, 3, 5, 7, 10, and 15 wt.%. The TiO_2_ paste with the electrospun nanorods was cast on pre-cleaned fluorine-doped tin dioxide (FTO; Pilkington TEC glass, 8 Ω cm^−2^, Pilkington Group Limited, St Helens, UK) using a squeeze printing method. The TiO_2_ films were sintered at 450°C for 30 min. The thickness of the TiO_2_ films was about 10 μm, and the active area of the TiO_2_ electrode was 0.25 cm^2^. The obtained TiO_2_ film was immersed in 0.5 mmol ethanol solution of N719 dye (Solaronix, Aubonne, Switzerland) for 24 h to adsorb the dye molecules. A Pt counter electrode was fabricated by squeeze printing of the Pt-Sol (Solaronix) on an FTO substrate. The sandwich-type solar cell was assembled by placing a Pt counter electrode on the dye-sensitized TiO_2_ electrode. The redox electrolyte (Dyesol) was injected between the electrodes.

### Characterization

An AM 1.5 solar simulator (white light from a 150-W Xenon lamp, McScience, Suwon-si, South Korea) was used as the light source. The incident light intensity was calibrated with a standard Si solar cell (Japan Quality Assurance Organization, Tokyo, Japan). Electrochemical impedance spectroscopy (EIS) was conducted using Iviumstat (Ivium Technologies B.V., Eindhoven, the Netherlands) at an open-circuit potential at frequencies ranging from 10^−1^ to 10^5^ Hz with an AC amplitude of 10 mV. The diffusion coefficients and electron lifetime of the electrons in the TiO_2_ films were determined using ModuLight-module under a red LED (*λ* = 625 nm) as light source (Ivium Technologies). The values of the diffusion coefficient and electron lifetime were obtained under 0.55-, 0.7-, 0.85-, and 1-V light intensity.

## Results and discussion

**Figure 1:**
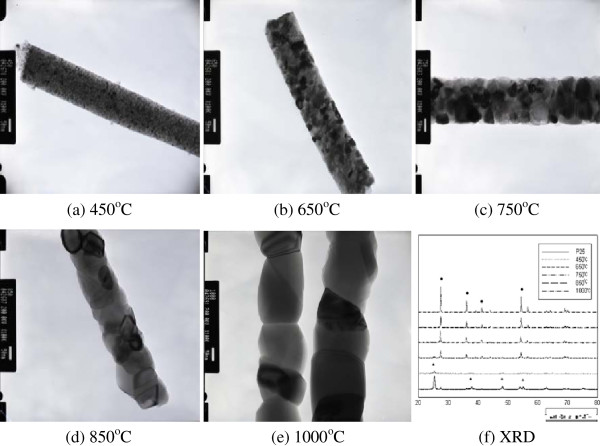
**TEM images and XRD data of TiO**_**2 **_**nanorods after sintering at various temperatures.** (**a**) 450°C, (**b**) 650°C, (**c**) 750°C, (**d**) 850°C, (**e**) 1,000°C, and (**f**) XRD.

TEM images and XRD data of the TiO_2_ nanorods sintered at various temperatures are shown in Figure 1. The phase transition of the TiO_2_ was observed depending on the sintering temperatures. With increasing sintering temperature, the amorphous TiO_2_ underwent phase transition to anatase and rutile structures. The crystallinity increased and the crystal size in the nanorods grew with increasing temperature. Comparison with the XRD peaks of P25, which contains both anatase and rutile phases, confirmed that the sintered nanorods at 750°C, 850°C, and 1,000°C had rutile peaks. During the high-temperature thermal treatment, the average crystal size increased, reducing the grain boundaries and crystal defects. The decreased number of trap sites on the nanorods reduced the number of obstacles on the fast electron moving paths. These effects influenced the charge trap conditions and consequently increased the electron diffusion speed [20]. Among the nanorods sintered at various temperatures, those sintered at 850°C had the highest energy conversion efficiency in DSSCs. The photoelectrodes using a homemade paste with P25 TiO_2_ and 3 wt.% nanorod sintered at 450°C, 650°C, 750°C, 850°C, and 1,000°C exhibited efficiencies of 3.32%, 3.12%, 3.16%, 3.47%, and 3.41%, respectively.

**Figure 2:**
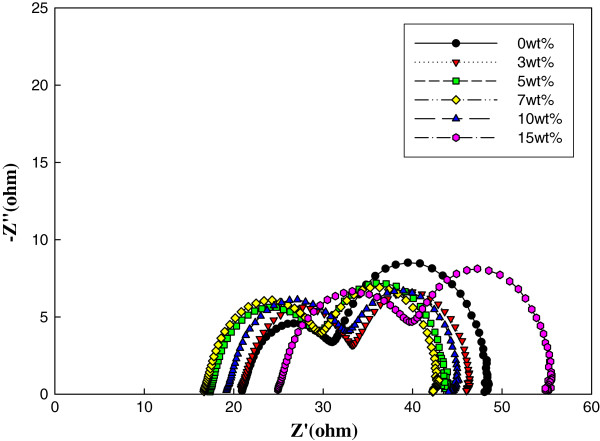
Impedance spectra of the cells with the rutile nanorods.

The internal resistance was investigated by EIS. The impedance spectra of the cells prepared using various amounts of nanorods sintered at 850°C are presented in Figure 2. The semicircles are related to the electron transfer resistance and the tendency of recombination at the TiO_2_/electrolyte interface [21]. The arc decreased with increasing amount of nanorods until 7 wt.% and then increased. The 1-D nanorods improved the charge transport and decreased electron recombination by providing fast moving paths for electrons. Although 1-D nanostructured nanorods have been proven to deliver a higher short-circuit photocurrent density (*J*_sc_) than TiO_2_ nanoparticles, too many large rutile nanorods could become a barrier for the electrons due to the higher energy level of the rutile phase.

**Figure 3:**
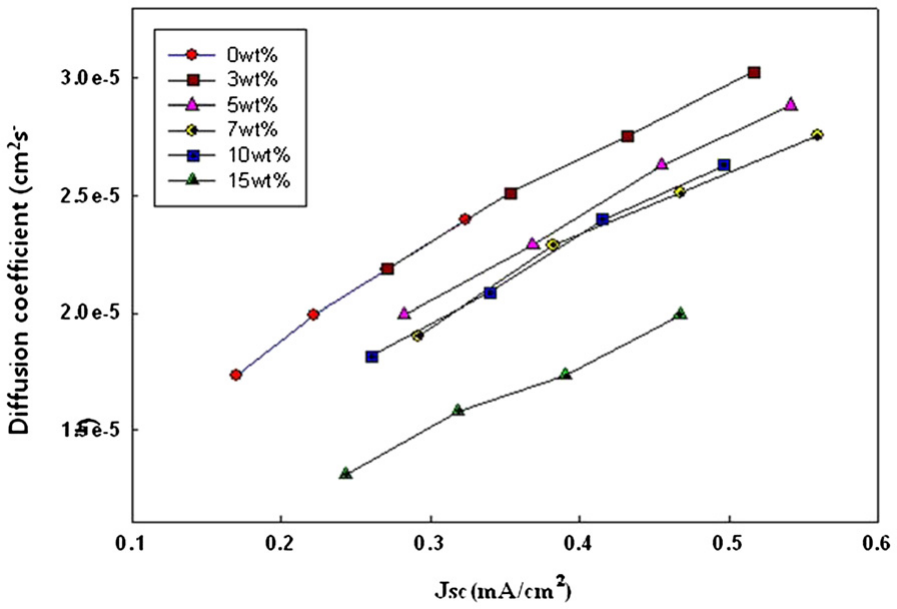
**Electron diffusion coefficients (*D***_**n**_**) for the DSSCs with the 1-D rutile nanorods.**

**Figure 4:**
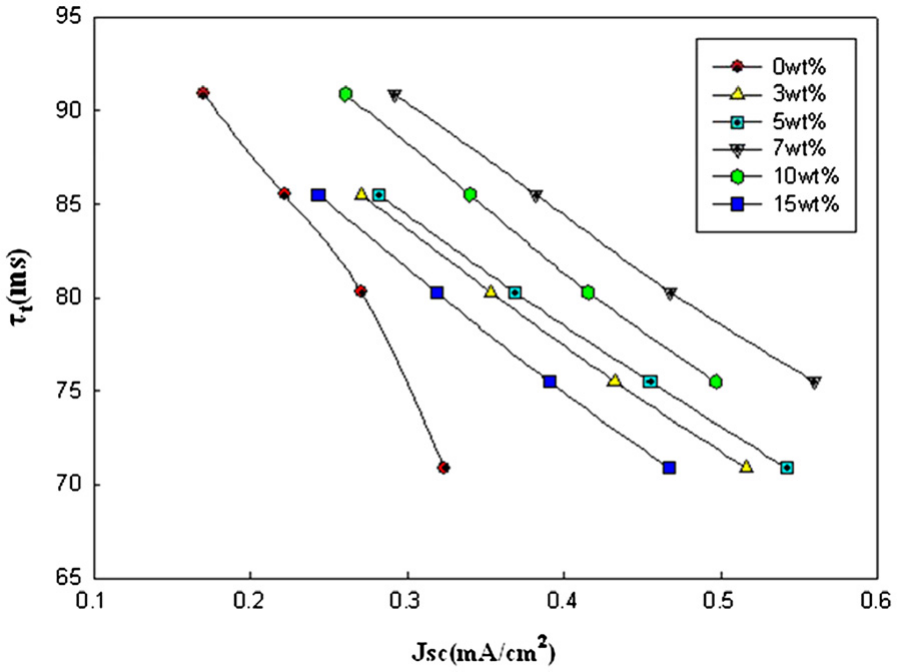
**Electron lifetimes (*τ***_**r**_**) for the DSSCs with the 1-D rutile nanorods.**

Figures 3 and 4 show the electron diffusion coefficients (*D*_n_) and lifetimes (*τ*_r_) of the rutile TiO_2_ nanorods as a function of *J*_sc_. The *D*_n_ and *τ*_r_ values were determined by the photocurrent and photovoltage transients induced by a stepwise change in the laser light intensity controlled with a function generator. The trends of diffusion coefficients by TiO_2_ structures are known to be reasonably consistent with the resistances in the TiO_2_ film determined by EIS [22,23]. In Figure 3, all the DSSCs with 1-D rutile nanorods have a higher *J*_sc_ than the 0 wt.% TiO_2_ electrode. Table 1 shows that the diffusion coefficients of the electrode with the 1-D rutile nanorods are higher than those of the electrode without the nanorods. However, the value of the diffusion coefficient at the electrode with 15 wt.% nanorods decreased due to the higher energy level of the rutile phase in the nanorods. In Figure 4, the *J*_sc_ of the electrode with the 1-D nanorods is also increased. The lifetime of the electrodes with rutile nanorods is relatively similar to the 0 wt.% electrode at 3, 5, and 15 wt.% and higher at 7 and 10 wt.%. The 1-D nanorods with the increased *τ*_r_ values can provide an electron pathway. The improved diffusion coefficient and the provided electron pathway result in a synergistic effect that increases the *J*_sc_.

**Table 1:** Diffusion coefficients and lifetime values of the DSSCs with 1-D rutile nanorods at 1-V light intensity

	**0 wt.%**	**3 wt.%**	**5 wt.%**	**7 wt.%**	**10 wt.%**	**15 wt.%**
Diffusion coefficient (cm^2^ s^−1^)	2.40E−05	3.03E−05	2.89E−05	2.76E−05	2.63E−05	1.99E−05
Lifetime (*τ*_r_) (ms)	70.9	70.9	70.9	75.5	75.5	70.9

Table 2 shows the performances of the DSSCs with the 1-D structured rutile nanorods. The *J*_sc_ value increased with increasing amount of nanorods until 10 wt.% and then decreased at 15 wt.%. The conversion efficiency of the cells using the rutile-phase nanorods was improved depending on the amount of nanorods. In the cells with nanorods, more electrons could move along the 1-D rutile nanorods due to the enhanced electron diffusion and the reduced electron recombination. Furthermore, the conversion efficiency was improved due to the enhanced electrolyte penetration. The electrolyte could easily penetrate into the photoelectrode due to the random packing of 1-D nanorods because of the porosity. The enhanced interpenetration of the electrolyte led to dye regeneration by redox process of the electrolyte and thus enhanced the energy conversion efficiency with improved photocurrent. As a result, the increased *J*_sc_ affected the enhancement of the energy conversion efficiency. However, the efficiency of the cell with 15 wt.% nanorods was decreased because the random distribution of a large number of rutile nanorods created a barrier to the electron transport due to the higher energy level of the rutile phase. An excessive amount of 1-D TiO_2_ nanorods can limit the DSSC performance.

**Table 2:** Cell performances of the DSSCs with the 1-D rutile nanorods

	**0 wt.%**	**3 wt.%**	**5 wt.%**	**7 wt.%**	**10 wt.%**	**15 wt.%**
*V*_OC_	0.71	0.72	0.74	0.73	0.74	0.74
*J*_SC_	10.55	11.97	11.32	12.29	11.13	10.07
Fill factor	63.17	61.71	69.38	68.52	69.43	67.24
Efficiency	4.75	5.35	5.79	6.16	5.68	4.99

## Conclusions

1-D rutile nanorods can provide a fast moving pathway for electrons and decrease electron recombination. In this study, the nanorods with high crystallinity showed enhanced energy conversion efficiency with reduced TiO_2_/electrolyte interface resistance. However, an excessive amount of randomly distributed rutile nanorods could create an obstacle to the moving electrons and reduce the internal surface area, even though they provided the electron moving paths. The charge-transfer resistance was decreased with increasing rutile nanorod loading up to 7 wt.%, but the electrical resistance was increased as the loading exceeded 10 wt.%. A 7 wt.% loading of 1-D rutile nanorods was considered the best condition for optimizing the performance of the DSSCs. The energy conversion efficiency of the optimized cell was 6.16%.

## Abbreviations

1-D: one-dimensional; DSSCs: dye-sensitized solar cells; EIS: electrochemical impedance spectroscopy; FTO: fluorine-doped tin dioxide; TEM: transmission electron microscopy; XRD: X-ray diffraction.

## Competing interests

The authors declare that they have no competing interests.

## Authors’ contributions

YHJ fabricated the DSSCs. KP and JSO performed the spectroscopic study. DK and CKH drafted the manuscript. All authors read and approved the final manuscript.
